# Soy intake and breast cancer: elucidation of an unanswered question

**DOI:** 10.1038/sj.bjc.6604182

**Published:** 2008-01-08

**Authors:** M D Gammon, B N Fink, S E Steck, M S Wolff

**Affiliations:** 1 Department of Epidemiology (CB No 7435), University of North Carolina, Chapel Hill, NC, USA; 2 Department of Public Health & Homeland Security, University of Toledo, Toledo, OH, USA; 3 Department of Epidemiology and Biostatistics, University of South Carolina, Columbia, SC, USA; 4 Department of Community and Preventive Medicine, Mt Sinai School of Medicine, New York, NY, USA

Breast cancer is the most commonly diagnosed cancer among women in many Western populations (American Cancer Society, 2004), although in Asian countries this disease is comparatively less frequent (Parkin *et al*, 1992). For example, breast cancer incidence rates historically have been four to seven times higher among women in the United States than those in China or Japan (Kelsey and Horn-Ross, 1993), yet the underlying reasons for this disparity are not entirely clear (Kelsey and Horn-Ross, 1993; Ziegler *et al*, 1993). The geographic variation in risk does not appear to be due to large genetic differences between ethnicities, because breast cancer incidence rates among Asian-American women shifts substantially toward those of white American women within a few generations after migration (Chie *et al*, 1995; Stanford *et al*, 1995; Wu *et al*, 1998).

These international variations in incidence rates coupled with the results from migration studies suggest breast cancer risk is influenced by environmental exposures, reproductive history, and lifestyle choices, including diet (Kelsey and Horn-Ross, 1993; Ziegler *et al*, 1993). Some researchers attribute the international heterogeneity in risk to the higher consumption of soy products in Asian countries (Ren *et al*, 2003). Since Americans and other Western populations tend to consume less flavonoid-rich foods compared to Asians and Asian-Americans (Dai *et al*, 2002), it is possible that compounds in these products with known biologic anti-cancer activity may decrease breast cancer risk and improve survival after diagnosis.

Flavonoids, of which soy-based isoflavones are the most intensely studied, lower endogenous hormone levels in pre- (Dai *et al*, 2002; Ren *et al*, 2003) and post-menopausal women (Murkies *et al*, 1995), and inhibit transcriptional factors involved in cancer metastasis (Valachovicova *et al*, 2004; Slivova *et al*, 2005), including vascular endothelial growth factor, which is strongly associated with tumour angiogenesis (Masuda *et al*, 2002). These findings provide support for a biologically plausible association between dietary intake of flavonoids, particularly isoflavones, on breast cancer risk and prognosis.

Unfortunately, the multiple investigations conducted to date among human populations to evaluate a possible soy–breast cancer link appear to be inconsistent and interpretation is not straightforward. The meta-analysis presented by Wu *et al* in this issue of *BMJ* was undertaken to obtain a summary effect estimate of the association in epidemiologic studies. Their approach brings clarity to the heated debate on this controversial issue.

One of the most important contributions of the meta-analysis undertaken by Wu *et al* is the separation of the epidemiologic studies conducted among Asian populations from those conducted among Western populations. This simple stratified approach elegantly demonstrates that a higher intake of isoflavones—about 20 mg per day or more as compared with a lower intake of about 5 mg per day—among Asian populations is associated with a consistent 29% reduction in the risk of developing breast cancer. Importantly, an inverse dose–response relation was evident; modest intake of about 10 mg per day of isoflavones was associated with a modest 12% decrease in breast cancer risk. Further, the risk reductions among Asian women were observed for both pre-and postmenopausal breast cancer. Moreover, the effect appeared to be stronger among those consuming soy during adolescence than among those who had only consumed soy as an adult, although research on early-life consumption patterns is sparse. In contrast, isoflavone intake of about 0.8 mg per day, which Wu *et al* reported as the higher level of consumption among Western populations (but which is obviously far below even the lower levels of intake for Asian populations), was not associated with breast cancer risk. In all, these exciting findings by Wu *et al* underscore the importance of considering exposure dose and timing when evaluating the effects of soy intake on breast cancer risk.

Other concerns have also contributed to the difficulty in interpreting the numerous population studies on the soy–breast cancer association, and again the thoughtful methodologic approach of Wu *et al* sheds light on these issues. For example, many of the investigations conducted to date have used the case–control study design, which may yield biased effect estimates due to possible differential recall of exposure based on the subjects' case–control status. However, as shown by Wu *et al*, results among Asian populations from both cohort and case–control studies yielded nearly identical summary effect estimates. Further, if recall bias was the underlying reason for the inverse association noted in the meta-analysis presented by Wu *et al*, then the risk reduction associated with isoflavone intake should have been evident among case–control studies conducted among Asian and Western populations, which was not the case. Thus, recall bias does not appear to be the reason for the observed risk reductions associated with soy intake among Asian populations.

An important issue that still remains unclear is whether any of the biologically active anti-cancer flavonoid compounds influence breast cancer risk among Western populations. Most previous epidemiologic studies have been hampered by focusing solely on intake of isoflavones, which are usually consumed by Westerners through intake of soy-based products such as processed foods. Challenges associated with assessing intake of isoflavones from these types of products could perhaps be overcome by utilising a biomarker of exposure, although single measures common to epidemiologic studies are not easily interpreted. Instead, it may be more productive to consider intake of a broader range of flavonoids, many of which occur in foods more frequently consumed by Western populations (such as tomatoes, green peppers, berries, and citrus fruits (Fink *et al*, 2006)). These other flavonoid classes can then be assessed using more conventional nutritional epidemiology methods (Fink *et al*, 2006). Recent studies conducted in Greece (Peterson *et al*, 2003), Italy (Bosetti *et al*, 2005), and the United States (Fink *et al*, 2007b) using this alternative approach consistently report a modest decrease in the risk of breast cancer in relation to intake of flavones. Thus, further exploration of the potential chemo-preventive effects of additional flavonoid classes on breast cancer appears warranted.

The review by Wu *et al* also highlights other understudied soy–breast cancer issues. In particular, the authors note that few investigators have formally evaluated the effects of soy intake on survival among women diagnosed with breast cancer. For example, one study conducted among a cohort of women with breast cancer in Shanghai (Boyapati *et al*, 2005) observed no association between consumption of isoflavones at diagnosis and subsequent mortality. In contrast, in a recent publication by our own group (Fink *et al*, 2007a) a reduction in all-cause and breast cancer-specific mortality was observed among a cohort of breast cancer survivors in the United States, who reported consuming higher levels of total flavonoids, and particularly flavones. To the best of our knowledge, no other reports on this subject have been published to date.

In sum, the meta-analysis by Wu *et al* notably demonstrate that relatively high intake of 10–20 mg per day of isoflavones, a consumption level frequently found among Asian populations, is inversely associated with a 10–30% reduction in risk of breast cancer. Lower isoflavone intake of <1 mg, a level more commonly consumed by Western populations, does not appear to have any beneficial effects on breast cancer risk. Additional research is needed to assess whether soy consumption that begins during the adult years is sufficient to decrease risk, or whether only intake patterns that were initiated during childhood affect the subsequent risk of developing breast cancer. In addition, clarification is needed on whether intake of other flavonoid classes, particularly those in foods such as fruits and vegetables more frequently consumed by Western populations, are associated with breast cancer risk reductions as has been shown for flavones in three recently published studies. Lastly, whether consumption of isoflavones or other flavonoid classes affects mortality among breast cancer survivors has been evaluated in only two previous reports, with conflicting results, and thus this issue also deserves to be more extensively evaluated.
